# External and Internal Work Load During a Mountain Time Trial in Trained Handcyclists Versus a World-Class Handcyclist and Determinants of Performance

**DOI:** 10.1097/PHM.0000000000002050

**Published:** 2022-05-17

**Authors:** Sonja de Groot, Ingrid Kouwijzer, Sven P. Hoekstra, Guido Vroemen, Linda J.M. Valent, HandbikeBattle group, Lucas H.V. van der Woude

**Affiliations:** From the Amsterdam Rehabilitation Research Center | Reade, Amsterdam, the Netherlands (SdG, IK); Department of Human Movement Sciences, Faculty of Behavioural and Movement Sciences, Vrije Universiteit, Amsterdam, the Netherlands (SdG); Research and Development, Heliomare Rehabilitation Center, Wijk aan Zee, the Netherlands (IK, LJMV); University of Groningen, University Medical Center Groningen, Center for Human Movement Sciences, Groningen, the Netherlands (IK, LHVvdW); Peter Harrison Centre for Disability Sport, School of Sport, Exercise and Health Sciences, Loughborough University, Loughborough, United Kingdom (SPH); Sports Medical Centre, Amersfoort, the Netherlands (GV); and University of Groningen, University Medical Center Groningen, Center for Rehabilitation, Groningen, the Netherlands (LHVvdW).

**Keywords:** Power Output, Heart Rate, Exercise Test, Handcycling Race

## Abstract

The aims of the study were to evaluate the external and internal work load of trained handcyclists during a mountain time trial, to compare the results with a world-class handcyclist, and to identify time trial performance determinants.

**Design:**

Ten trained and one world-class handcyclists performed a graded exercise test to determine power output and heart rate at the (first and second) ventilatory thresholds and exhaustion. Power output and heart rate were continuously measured during the race.

**Results:**

The mean absolute power output during the race (119 ± 21 vs. 203 W, *P* < 0.001) was lower in the trained handcyclists compared with the world-class handcyclist. The absolute and relative heart rate during the race (86 ± 7% vs. 88%, *P* = 0.40) and relative power output during the race (66 ± 10% vs. 62%, *P* = 0.24) were similar. Trained handcyclists cycled significantly less time at a power output between first and second ventilatory thresholds (48% vs. 64%, *P* = 0.02) and more at a power output greater than second ventilatory threshold (34% vs. 11%, *P* = 0.005). Power output at the second ventilatory threshold showed the strongest correlation with finish time (*r* = −0.78) and peak power output with mean power output of the race (*r* = 0.90).

**Conclusions:**

The laboratory outcome peak power output and power output at the second ventilatory threshold are important performance determinants for longer time trials in handcyclists, and it is, therefore, important to improve these outcomes with training. Because the trained handcyclists cycled most of the race in intensity zones 2 and 3, it is recommended to incorporate these zones also in the training.

**What Is Known**

- The HandbikeBattle is mountain time trial in Austria in which former patients of rehabilitation centers participate. Although there are studies on handcycling in the rehabilitation setting and competitive sport domain, the physical demands of climbing a mountain in a handcycle are yet unclear.

**What Is New**

- This study shows that power output at the second ventilatory threshold and at exhaustion are important performance determinants for mountain time trials in handcyclists. Because the trained handcyclists cycled most of the race in intensity zones 2 and 3, it is recommended to incorporate these zones also in the training.

The HandbikeBattle is a yearly organized mountain time trial (20.2 km, 839-m elevation) in Austria in which former patients of Dutch rehabilitation centers participate.^1^ These former patients are selected by their rehabilitation centers because they might need this challenge to become mentally and/or physically fit. Some of the HandbikeBattle participants discover that they are really talented in handcycling and start to participate in competitive handcycling events and eventually in the Para-cycling World Championships and Paralympic Games.

Although there is an emerging body of evidence regarding handcycling in the rehabilitation setting^2^ and in the competitive sport domain,^3^ the mechanical and physiological demands of climbing a mountain in a handcycle are yet unclear. A previous study^1^ investigated the exercise intensity distribution of 17 handcyclists with spinal cord injury (SCI) during this HandbikeBattle time trial. Results showed that the HandbikeBattle participants exercised most of the time at a vigorous intensity (73%),^1^ which is in contrast to the low to moderate intensities for level ground marathons and/or ultra-long handcycling races.^1,4,5^

Despite the linear relationship between internal load measures such as heart rate (HR) and external load measures such as power output (PO) in handcycling,^6^ HR can be influenced by environmental conditions, cardiovascular drift, the VO_2_ slow component,^7^ and, among people with SCI, also by impairment of the autonomic nervous system.^8^ While PO is often an explicit outcome in individual laboratory-based peak handcycle performance tests, both absolute and relative indicators of PO and HR allow a description of the external and internal load during race events. Power output is often derived from specific sensor devices and is deemed to be the best objective indicator of external load because it takes both velocity and mechanical resistance forces (i.e., force, due to for example wind, slope, body mass) into account. The PO distribution has been studied during races in professional cycling.^9–11^ From these studies, it is clear that PO distribution is different in time trials (mostly in high-intensity PO zone) compared with mass start stages (mostly in low-intensity PO zone)^9^ and that the absolute PO is similar between flat and semimountainous terrain but higher in mountainous terrains.^11^ Furthermore, it was shown that compared with PO, HR underestimated the time spent at the low- and high-intensity zones and overestimated time spent in the moderate intensity zone.^10^ While the power profile has been investigated extensively in road cycling, current research on the PO demands of handcycling outside the laboratory is limited to level ground handcycling,^4,5,12^ which leaves a gap regarding preparation for uphill handcycling races. Last, not much is known about the PO determinants of a handcycling time trial performance. Previous studies found that peak aerobic PO,^13,14^ aerobic lactate threshold,^14^ and PO at 4 mmol/l^13,14^ are important determinants of a 15- to 16-km handcycling time trial performance in a laboratory^14^ or on a cycling racing circuit.^13^

To get more insight in the mechanical and physiological demands of uphill handcycling, the HR and PO of trained handcyclists during the HandbikeBattle mountain time trial were investigated. For a good interpretation, these results were compared with a world-class (WC) handcyclist who was familiar with the HandbikeBattle route. The aims of this study were, therefore, (1) to investigate the external and internal work load of trained handcyclists and compare their results with a WC handcyclist (the “criterion standard”); (2) to investigate the external and internal work load during three sections (flat, semimountainous, mountainous) of the HandbikeBattle; and (3) to identify the most important PO determinants of this time trial performance.

## METHODS

### Participants

People with lower-limb disabilities are allowed to compete in the HandbikeBattle if they have no contraindications for physical exercise. This was checked by a mandatory medical screening including a graded exercise test (GXT). Because of a limited availability of the expensive power meters, a device on a (hand)cycle that measures the PO of the rider in real life, a subgroup (*n* = 36) was selected to participate in the present study. This subgroup was selected based on having a peak handcycling PO (POpeak) greater than 100 W in the GXT at the medical screening, trained relatively often, and were motivated to handcycle with a power meter during all training sessions and the race. Exclusion criteria for the study were: not being able to come to the rehabilitation center in the month before the race for an additional GXT, not owning a handcycle in which a wheel with power meter could be fitted, and not understanding the Dutch language.

The male WC handcyclist (H4) was an international medalist at major global championships,^15^ had a lower-limb disability, competes in an arm-powered handcycle, and was familiar with the HandbikeBattle route.

All HandbikeBattle participants were classified by official classifiers before the HandbikeBattle and following the classification rules from the Union Cycliste Internationale.^16^

This study was approved by the local ethics committee of the Centre for Human Movement Sciences, University Medical Centre Groningen, the Netherlands (ECB/2012_12.04_l_rev/Ml). All participants signed an informed consent before participating in this study.

### Design

Power output and HR were measured during a laboratory-based GXT and during the HandbikeBattle race. Data were collected in the month before (laboratory) and during five editions (2015–2019) of the HandbikeBattle. The race was held each year in June on the Kaunertaler Gletscherstrasse in Austria, starting at the toll gate and ending at the Ochsenalm.^1^

### Graded Exercise Test

#### Trained Handcyclists

In the month before the HandbikeBattle, the selected handcyclists conducted the GXT. The GXT was performed in the participant’s own handcycle, which was attached to a Cyclus2 ergometer (Cyclus2; RBM elektronikautomation GmbH, Leipzig, Germany). During the GXT, breath-by-breath data were collected using a COSMED K4B2 (COSMED, Rome, Italy) in 2015 and 2016, an Oxycon Mobile (Oxycon Mobile; Carefusion, Höchberg, Germany) in 2017 and a Cortex (CORTEX Biophysik, Leipzig, Germany) in 2018 and 2019. Heart rate was measured continuously using a Polar HR sensor chest strap (Polar Heart rate sensor; Polar Electro Oy, Kempele, Finland).

The participant started the GXT with a 5-min warm up at a self-selected cadence and PO. Thereafter, the participant had 2-min rest, followed by incremental 3-min stages at a self-selected cadence greater than 60 revolutions per minute (rpm). With every 3-min block, the PO increased with 20 W until volitional exhaustion. The starting PO was determined as the achieved POpeak during the first GXT, performed during the medical screening, minus 120 W. If the participant had a POpeak of 120 W or lower at the first GXT, the PO started at 30 W and increased with 10 W every 3 mins.

#### World-Class Handcyclist

The GXT of the WC athlete was performed in the athlete’s own handcycle using a Cyclus2 ergometer. Breath-by-breath and HR data were collected using a COSMED Quark CPET (COSMED, Rome, Italy). The starting PO was set at 100 W with an increase of 15 W per 3 mins at a cadence approximately 85 rpm. When the second ventilatory threshold (VT2) was reached, the PO increased with 15 W every minute until volitional exhaustion.

#### Data Analyses Laboratory Test

From the GXT, the first ventilatory threshold (VT1) and VT2, POpeak, and HRpeak were determined as described in detail by Kouwijzer et al.^17^ Breath-by-breath data were filtered with a moving average filter over 15 breaths before the analysis. Three plots were presented to an experienced rater to determine VT1 and VT2: (1) VCO_2_ versus VO_2_, (2) the ventilatory equivalents of oxygen (Ve/VO_2_) and carbon dioxide (Ve/VCO_2_) versus time, and (3) respiratory exchange ratio versus time.

The PO accompanied with this time that VT1 and VT2 occurred was determined as the highest PO maintained for 3 mins with an addition of 3.3 W for every 30 secs maintained in the block in which the VT occurred (1.7 W for the 10-W protocol). The HR and VO_2_ at the time of VT1 and VT2 were determined as the 15-breaths-average value at the VT.

The POpeak (in watts) was defined as the highest PO maintained for 3 mins during the GXT with an addition of 3.3 W for every 30 secs maintained in the final block (1.7 W for the 10-W protocol).^18^ VO_2_peak (in liters per minute) and HRpeak (in beats per minute [bpm]) were defined as the highest 15-breath average of VO_2_ and HR, respectively.

### Measurements During the HandbikeBattle

The PO during the race was measured with a PowerTap P1 Hub system (PowerTap; Saris Cycling Group, Madison, WI; typical error: 1.5%). The PowerTap (sample frequency: 1 Hz) and a Garmin HR monitoring chest strap were connected to a Garmin Edge 500 GPS system (Olathe, KS).

The WC handcyclist used a Quarq Dzero crank powermeter (Quarq, Spearfish, SD), a Garmin 1000 Edge GPS system (Garmin, Schaffhausen, Switzerland), and a Tacx HR monitoring chest strap (Tacx, Wassenaar, the Netherlands). Together, this equipment enabled the registration of PO, speed, traveled distance, HR, and cadence at every second throughout the HandbikeBattle.

#### Data Analyses Race

Before race data analysis, outlier values of PO above 2*POpeak and HR below the HR at rest were replaced by the average of the values before and after the data point, because they are thought to be unrealistic, that is, erroneous values. The total time the participant spent on stops during the race (%time stopped) was calculated as the time that both speed and cadence were 0 (in meters per second or rpm, respectively), which was divided by the total race time. Furthermore, data points where the speed was 0 m/sec but cadence greater than 0 rpm were considered errors and not used for further analysis. When, as a result, a data set had too many missing data (>10% of the data), this data set/participant was excluded from the study.

The valid race data were used to determine the mean and peak values for HR (in bpm), PO (in watts), cadence (in rpm), and velocity (v in meters per second). The mean and peak HR and PO were also expressed as percentage of the peak value achieved during the GXT.

The VT1 and VT2 determined in the GXT were used to calculate three PO and HR zones.^10^ In zone 1, the PO or HR during the race is below the PO or HR at VT1 (low intensity). In zone 2, the PO or HR during the race lies in between the PO or HR at VT1 and VT2 (moderate intensity), that is, lactate begins to accumulate in the blood and the breathing rate begins to increase. In zone 3, the HR or PO during the race is above the PO or HR at VT2 (high intensity), that is, lactate quickly accumulates in the blood and the person needs to breathe heavily. The amount of time spend in every HR and PO zone during the race was calculated as a percentage of the total race time.

**FIGURE 1:**
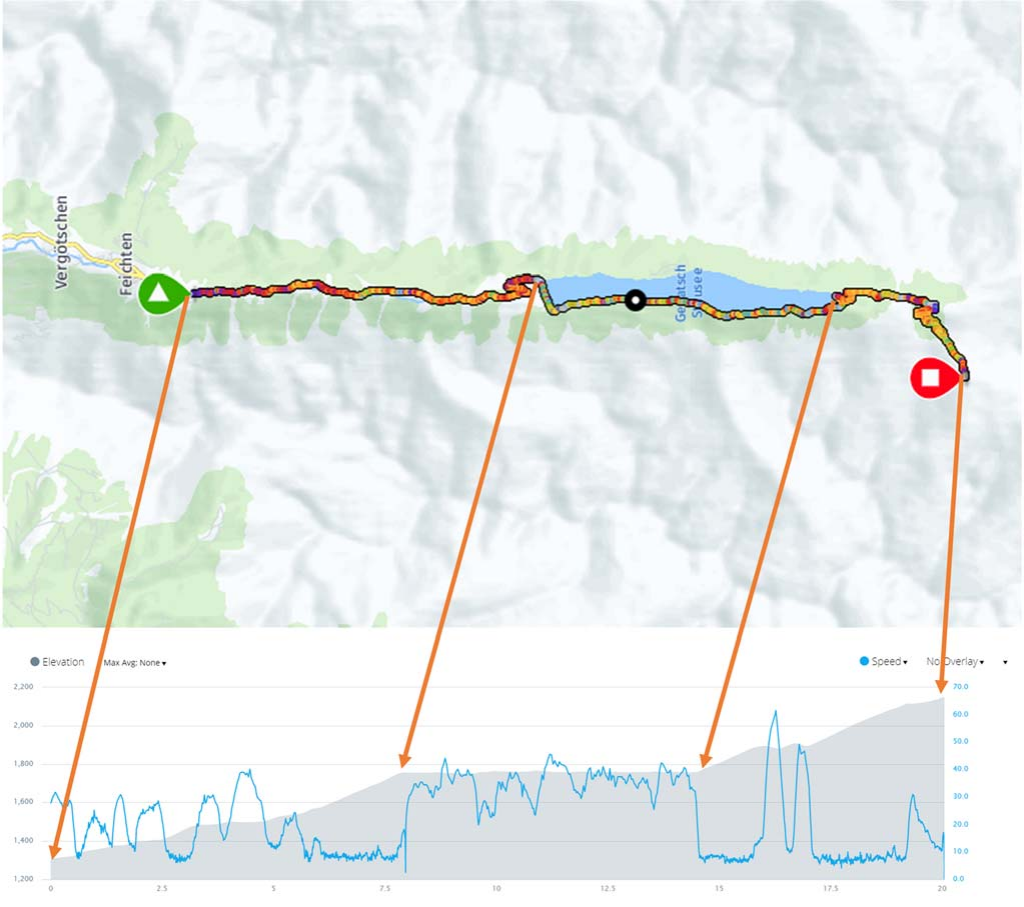
HandbikeBattle route, elevation, and speed profile of a typical example.

Furthermore, the race data were cut into three sections based on the race trajectory, using the GPS data recorded by the Garmin device. Figure 1 shows the HandbikeBattle route, elevation profile, and the three sections. The first section (semimountainous), from the start until the reservoir, has a length of 8.0 km and an altitude gain of 446 m. The second section (flat), around the reservoir, has a length of 6.6 km and 4-m altitude gain. The third section (mountainous), from the end of the reservoir until the finish, has a length of 5.5 km and a total altitude gain of 389 m. This third section contains two short descents and many steep uphill hairpin turns. All calculations performed on the total race were repeated for these three sections. All calculations were executed by custom-written programs in MATLAB 2019a (Mathworks, Natic, MA) script.

### Statistics

First, potential differences in personal characteristics and GXT outcomes between the group with complete PO data (study participants) and the group with incomplete PO and/or HR data (nonstudy participants) during the time trial were tested with an independent *t* test.

Descriptives were calculated for the outcomes of the GXT, the total race, and the three sections of the race. The one-sample *t* test was used to test for differences between the trained handcyclists and the WC handcyclist.

A repeated measures analysis of variance (within-subject factor: section) was used to determine whether there was a difference between sections in PO and HR outcomes within the trained handcyclists. Bonferroni tests were performed as post hoc analyses.

Pearson correlation coefficients were calculated between finish time and the PO outcomes of the laboratory test and during the time trial to identify the most important PO determinants of the time trial performance.

All statistical analyses were performed using SPSS (IBM SPSS Statistics 26; SPSS, Inc, IBM Corp, Armonk, NY). Values were considered significant at *P* < 0.05. Effect sizes (Cohen *d*) were calculated and classified as small when 0.20, medium when 0.50, and large when 0.80.^19^

## RESULTS

Thirty-six handcyclists received all the measurement equipment. Twenty five of them collected data during the race. However, six of them did not have PO data in their file; for three handcyclists, the data seemed to be incorrect (PO and velocity were zero while the altitude increased and the HR was not decreasing); and five handcyclists had too many missing PO data (>10%). Therefore, data of 11 handcyclists (1 WC and 10 trained handcyclists) were analyzed. One of the trained handcyclists only collected PO but no HR data during the race. Descriptives of the 10 trained handcyclists, all male, are shown in Table 1. The excluded (i.e., nonstudy) participants comprised 20 men and 6 women and had a lower POpeak (*P* < 0.001), VO_2_peak (*P* = 0.001), and slower finish time (*n* = 24, 3:50:32 ± 1:07:55, *P* < 0.001) compared with the study participants (Table 1).

**TABLE 1:** Personal and fitness characteristics and race outcomes of all 10 trained handcyclists included in the exercise profile analysis and compared, as a group, with the nonstudy participants

	Age, yr	Height, m	Body Mass, kg	Diagnosis	Classification	Handcycle	PO_peak_, W	PO_peak_, W/kg	VO_2peak_, l/min	VO_2peak_, ml/min/kg	HR_peak_, bpm	Finish Time, h:mm:ss	PO_mean_ Race, W	HR_mean_ Race, bpm
Study participants														
1	56	1.77	69.3	SCI L2 AIS C	H5	ATP	167	2.41	3.10	44.7	169	1:57:35	128	156
2	35	1.91	70.0	SCI Th11 AIS D	H3	AP	200	2.86	3.04	43.5	185	1:57:28	133	162
3	24	1.60	56.4	Cerebral palsy	H3	AP	160	2.84	2.51	44.5	194	3:31:23	77	138
4	31	1.85	72.4	SCI Th4 AIS A	H3	AP	183	2.53	2.30	31.8	181	2:28:51	100	145
5	55	1.73	74.2	Transfemoral amputation	H5	ATP	142	1.91	2.38	32.1	190	3:09:54	117	159
6	26	1.80	74.2	Paralyzed right lower leg	H4	AP	170	2.29	2.84	38.3	197	2:47:50	105	-
7	37	1.88	71.8	SCI L1 AIS C	H4	AP	190	2.65	3.37	47.0	175	2:03:55	122	164
8	43	1.93	84.8	SCI Th6 AIS A	H3	AP	227	2.68	4.76	56.1	191	2:08:20	149	169
9	28	1.90	82.2	Cerebral palsy and multitrauma	H4	AP	203	2.47	3.52	42.8	186	2:08:40	140	173
10	42	1.81	70.0	SCI L1 AIS A	H3	AP	165	2.36	2.61	37.3	182	2:08:22	116	15
Mean (SD)	38 (11)	1.82 (0.10)	72.5 (7.7)				181 (25)	2.50 (0.28)	3.04 (0.73)	41.8 (7.3)	185 (9)	2:26:13 (00:32:51)	119 (21)	157 (11)
Nonstudy participants (*n* = 26)												(*n* = 24)		
Mean (SD)	38 (15)	1.76 (0.09)	75.6 (15.4)				140 (29)*^a^*	1.87 (0.30)*^a^*	2.27 (0.49)*^a^*	30.1 (4.2)*^a^*	178 (13)	3:50:32 (1:07:55)*^a^*		

*^a^* Significant difference study participants and nonstudy participants (*P* < 0.05).

AP, arm-powered handcycle; ATP, arm-trunk-powered handcycle.

### Graded Exercise Test

Table 2 shows the GXT outcomes for the trained and WC handcyclists. Although the HR at the VTs and the HRpeak were similar between the trained and WC handcyclists, the VO_2_peak and POpeak as well as the VO_2_ and PO at the VTs were significantly lower for the trained handcyclists.

**TABLE 2:** Comparison of the exercise profile during the GXT and the total race between the trained (*n* = 10) and WC (*n* = 1) handcyclists

	Trained Handcyclists	
	*n*		WC Handcyclist	*P*	Cohen *d*
Graded Exercise Test					
PO VT1, W	10	83 (34)	183	<0.001	−2.9
PO VT1, W/kg	10	1.12 (0.41)	2.45	<0.001	−3.2
PO VT2, W	10	137 (24)	250	<0.001	−4.7
PO VT2, W/kg	10	1.88 (0.26)	3.35	<0.001	−5.7
POpeak, W	10	181 (25)	325	<0.001	−5.8
POpeak, W/kg	10	2.50 (0.28)	4.36	<0.001	−6.6
HR VT1, bpm	10	128 (20)	121	0.331	0.4
HR VT2, bpm	10	158 (10)	163	0.136	−0.5
HRpeak, bpm	10	185 (9)	187	0.482	−0.2
VO_2_ VT1, l/min	10	1.68 (0.53)	2.93	<0.001	−2.4
VO_2_ VT2, l/min	10	2.30 (0.41)	3.86	<0.001	−3.8
VO_2_peak, l/min	10	3.04 (0.73)	4.69	<0.001	−2.3
Race					
Finish time, h:min:s	10	2:26:13 (00:32:51)	01:19:00	<0.001	2.1
%Time stopped, %	10	5% (9)	0%	0.120	0.6
Cadence mean, rpm	10	63 (13)	81	0.002	−1.4
Mean velocity, km/h	10	9.3 (0.9)	15.2	<0.001	−6.6
POmean, W	10	119 (21)	203	<0.001	−4.0
POmean, W/kg	10	1.63 (0.19)	2.72	<0.001	−5.7
%POmean of POpeak, %	10	66% (10)	62%	0.238	0.4
POpeak race, W	10	320 (50)	533	<0.001	−4.3
%POpeak race of POpeak test	10	177% (19)	164%	0.055	0.7
%Time in PO zone 1, %	10	18% (14)	25%	0.160	−0.5
%Time in PO zone 2, %	10	48% (19)	64%	0.023	−0.8
%Time in PO zone 3, %	10	34% (20)	11%	0.005	1.2
HRmean, bpm	9	157 (11)	164	0.116	−0.6
%HRmean of HRpeak, %	9	86% (7)	88%	0.403	−0.3
HRpeak race, bpm	9	179 (12)	183	0.382	−0.3
%HRpeak race of HRpeak test	9	98% (5)	98%	0.850	0
%Time in HR zone 1, %	9	11% (20)	0%	0.152	0.6
%Time in HR zone 2, %	9	32% (31)	53%	0.083	−0.7
%Time in HR zone 3, %	9	57% (37)	47%	0.446	0.3

### Trained Versus WC Athlete

The exercise profile of the total race is shown in Table 2. Seven trained handcyclists stopped less than 1% of the race time, while the three others stopped 5%, 18%, and 27% of the total race time. The fastest trained handcyclists finished the race 38 mins later than the WC handcyclist. While the race velocity, absolute PO, and cadence were lower in the trained handcyclists, the absolute and relative HR and relative PO were similar between the trained and WC handcyclists. On average, the trained handcyclists cycled significantly less in PO zone 2 and more in zone 3 compared with the WC handcyclist while no significant differences were found for time in HR zones.

### Race Sections

**FIGURE 2:**
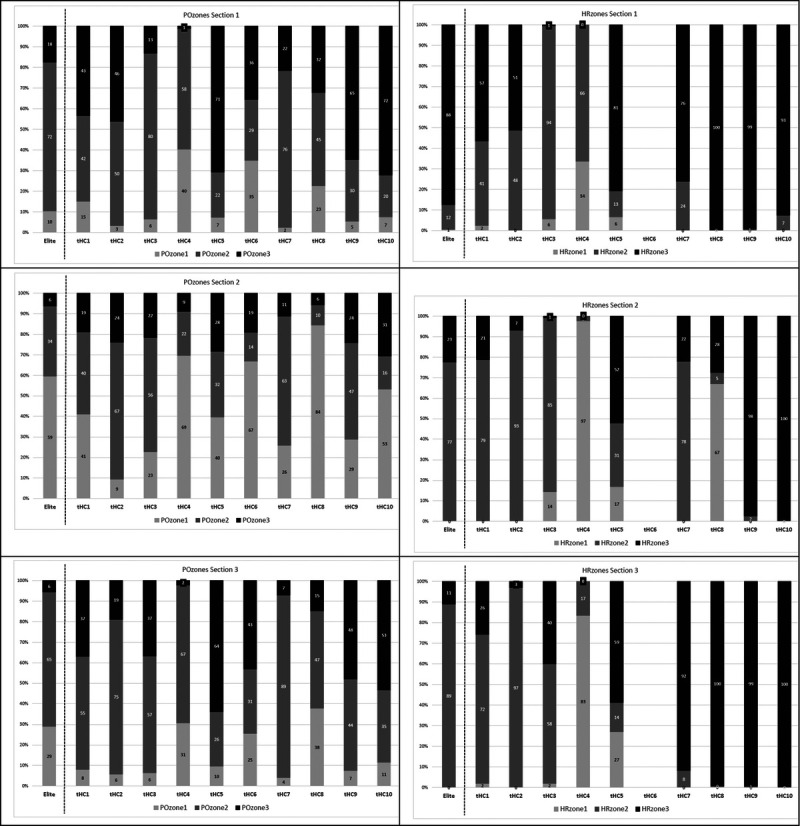
Percentage of the race time spent in different PO (left graphs) or HR (right graphs) zones for the elite athlete and 10 trained handcyclists during the three sections. The zones are categorized as: zone 1: HR or PO lower than the value at VT1; zone 2: HR or PO in between VT1 and VT2; zone 3: HR or PO higher than the value at VT2. The sections are: (1) semimountainous, (2) flat, and (3) mountainous.

Table 3 shows the exercise profile for the three different sections of the race. All outcome measures, except for %time in HR zones 1 and 2, showed a significant difference between the three race sections within the trained handcyclists. Post hoc tests showed that the mean velocity was higher and the POmean (in watts, watts per kilogram, and %POpeak) was lower during the flat section compared with the (semi)mountainous sections. On average, the trained handcyclists cycled more in PO zone 1 and less in PO zone 3 during the flat section compared with the other two sections. The HRpeak was lower during the flat section compared with the (semi)mountainous sections. As can been seen in Figure 2, the time spent in the PO and HR zones varied considerably among handcyclists.

**TABLE 3:** Comparison of the exercise profile during the three sections of the race between the trained (*n* = 10) and WC (*n* = 1) handcyclists as well as the comparison of the three race sections within the trained handcyclists

		Trained Handcyclists										WC Athlete		
Section		1			2			3				1	2	3
Distance, km		7.97			6.55			5.51			Within	7.97	6.55	5.51
Altitude gain, m		446			4			389			Trained HC	446	4	389
	*n*	Mean (SD)	*P* vs. elite	*d*	Mean (SD)	*P* vs. elite	*d*	Mean (SD)	*P* vs. elite	*d*	*P*			
Cadence mean, rpm	10	68 (12)^2,3^	0.002	−1.3	57 (15)^1^	<0.001	−2.3	60 (14)^1^	0.007	−1.1	0.001	84	91	75
Mean velocity, km/h	10	8.1 (1.1)^2,3^	<0.001	−5.1	24.3 (3.2)^1,3^	<0.001	−3.1	6.4 (0.5)^1,2^	<0.001	−7.2	<0.001	13.7	34.2	10.0
PO_mean_, W	10	126 (27)^2^	<0.001	−3.5	90 (17)^1,3^	<0.001	−4.5	120 (17)^2^	<0.001	−4.5	<0.001	221	166	196
PO_mean_, W/kg	10	1.72 (0.27)^2^	<0.001	−4.6	1.24 (0.22)^1,3^	<0.001	−4.5	1.66 (0.14)^2^	<0.001	−6.9	<0.001	2.97	2.22	2.63
%PO_mean_ of PO_peak_, %	10	70% (13)^2^	0.710	0.2	50% (7)^1,3^	0.658	−0.1	67% (9)^2^	0.032	0.8	<0.001	68%	51%	60%
%Time POzone 1, %	10	15% (13)^2^	0.328	0.3	45% (24)^1,3^	0.080	−0.6	15% (12)^2^	0.004	−1.3	<0.001	10%	59%	29%
%Time POzone 2, %	10	45% (21)	0.003	−1.3	36% (21)^3^	0.714	0.1	52% (20)^2^	0.083	−0.7	0.022	72%	34%	65%
%Time POzone 3, %	10	40% (24)^2^	0.017	0.9	19% (8)^1^	0.001	1.6	33% (21)	0.003	1.3	0.001	18%	6%	6%
HR_mean_, bpm	9	158 (14)	0.049	−0.8	151 (13)	0.044	−0.8	159 (10)	0.704	−0.1	0.027	169	161	160
%HR_mean_ of HR_peak_, %	9	86% (9)	0.230	−0.4	82% (8)	0.230	−0.5	87% (6)	0.814	0.2	0.024	90%	86%	86%
HR_peak_ race, bpm	9	173 (10)	0.015	−1.0	166 (9)^3^	0.501	−0.2	177 (13)^2^	0.246	0.4	0.021	183	168	172
%HR_peak_ race of HR_peak_ test, %	9	94% (5)	0.051	−0.8	90% (6)^3^	0.822	0	97% (6)^2^	0.042	0.8	0.018	98%	90%	92%
%Time HRzone 1, %	9	5% (11)	0.178	0.5	22% (36)	0.106	0.6	13% (28)	0.209	0.5	0.138	0%	0%	0%
%Time HRzone 2, %	9	33% (32)	0.093	0.7	42% (41)	0.032	−0.9	30% (36)	0.001	−1.6	0.480	12%	77%	89%
%Time HRzone 3, %	9	62% (39)	0.080	−0.7	37% (39)	0.324	0.4	58% (42)	0.010	1.1	0.036	88%	23%	11%

Note: section 1 = from start to reservoir (semimountainous); section 2 = along the reservoir (flat); section 3 = end reservoir to finish (mountainous).

Supercript 1, 2, and 3 refer to significant difference with that specific section number.

HC, handcyclist.

### Determinants of Finish Time

Moderate to strong associations were found between the PO laboratory outcomes and the finish time (Table 4), with the PO at VT2 showing the strongest relationship (*r* = −0.775). The correlations between PO during (sections of) the race and finish time showed slightly higher values (Table 4), with the POmean during the flat part showing the strongest correlation (*r* = −0.841) with the finish time. The outcomes of the laboratory test were correlated strongest with the POmean during the mountainous part with POpeak showing the highest correlation (*r* = 0.901).

**TABLE 4:** Correlations among the PO outcomes during the laboratory test, during the total time trial and different sections of the time trial and the finish time

	PO Laboratory Test			POmean Mountain			
	POpeak Laboratory, W	PO VT1, W	PO VT2, W	Total Race	Semimountainous	Flat	Mountainous
Finish time	−0.727*^a^*	−0.637*^a^*	−0.775*^b^*	−0.822*^b^*	−0.819*^b^*	−0.841*^b^*	−0.773*^b^*
POpeak laboratory, W	—	0.752*^b^*	0.957*^b^*	0.889*^b^*	0.861*^b^*	0.883*^b^*	0.901*^b^*
PO VT1, W	0.752*^b^*	—	0.786*^b^*	0.793*^b^*	0.761*^b^*	0.667*^a^*	0.847*^b^*
PO VT2, W	0.957*^b^*	0.786*^b^*	—	0.874*^b^*	0.850*^b^*	0.831*^b^*	0.889*^b^*

Correlations are based on 11 handcyclists.

*^a^* Correlation is significant at the 0.05 level (2-tailed).

*^b^* Correlation is significant at the 0.01 level (2-tailed).

## DISCUSSION

In summary, the trained athletes seem to pace the race differently compared with the WC athlete, that is, the WC handcyclist was able to cycle a larger percentage of the time in a less intensive PO zone (zone 2 instead of 3). In addition, PO at VT2 showed the strongest correlation with the finish time while POpeak showed a very strong correlation with POmean during the mountainous part of the race.

### Trained Versus WC Athlete

Although the absolute POmean during the race was lower in the trained athletes, the relative PO (as %POpeak) was comparable with the WC athlete. The WC athlete was able to produce more PO at a similar HR when compared with the trained handcyclists, indicating that the WC athlete’s body is operating more efficiently because of central and peripheral adaptations. The physical capacity of the WC handcyclist is, of course, related to the higher race PO and shorter race time. However, the longer race time in the trained handcyclists also has an effect on the power-duration curve. A previous study indicated that the mean PO over the last 30-sec of a 3-min all-out test decreased by 8% after 2 hrs of heavy-intensity exercise.^20^ Thus, the longer race duration itself might also have an effect on the mean PO that can be maintained and subsequently on the distributions in PO zones.

### Race Sections

The POmean was significantly lower on the flat terrain compared with the other two sections, which was also found in elite cyclists.^9,11^ This could have different causes but is, as far as known, not well studied. For example, the angle of attack on the cranks is different between a grade and level ground, and on a climb, you cannot have pauses in power production in contrast to flat sections. Furthermore, air resistance and gravitational resistances will be different during flat or mountainous sections. Of course, the mass of the athlete and handbike has a more important role in the (semi)mountainous sections, that is, more mass will require more external PO to produce the same velocity. Furthermore, a decrease in cadence during the steeper sections was also found before in cycling.^11^ The trained handcyclists had a lower cadence to climb the mountain compared with the WC handcyclists, with the largest difference during the flat section (57 vs. 91 rpm). A previous cycling study^21^ showed similar results, that is, that less-trained noncyclists chose lower cadences (65–80 rpm) at all standardized POs than trained cyclists (90–100 rpm). The authors^21^ speculated that less-trained noncyclists, who cycled at a higher percentage of VO_2_max, may have selected lower preferred cadence to reduce aerobic demand. This could also be the explanation for the lower cadence in our trained handcyclists.

During all three sections, the trained handcyclists spent more time in PO zone 3 (18%–38%) compared with the WC handcyclist (6%–18%). During the flat part, the trained handyclists cycled mainly in PO zone 1, while in the other sections, they cycled mainly in PO zone 2. A similar pattern was seen for the WC handcyclist. During flat cycling stages, up to 80% is spent in PO zone 1 and this percentage decreases slightly in semimountainous (76%) and mountain stages (69%).^9^ However, when cycling a time trial, the elite cyclists perform mainly in zone 3 (approximately 65%).^9^ Although our race was a time trial, our handcyclists were cycling most of the race in PO zone 2. This might be explained by the mountainous character and/or the duration of the HandbikeBattle time trial, which was much longer than the cycling time trial (38 mins) in Sanders et al.^9^

The percentage time in the exercise intensity zones is quite different within the handcyclists when it is based on PO or HR (Fig. 2). Some of the participants (no. 7–10) were (almost) continuously riding in HR zone 3, while, based on their PO data, this did not seem to be the case. Thus, it seems that the description of exercise intensity through HR tends to overestimate the time spent in zone 3 for some of the participants. These differences between exercise distribution in three zones based on HR and PO zones have been previously described in cycling.^10^ One of the reasons for this might be that the regulation of the cardiovascular system is slower to adapt to the quick changes in high and low PO.^10^ During descents, for instance, HR can still be in zone 3 while PO is already in zone 1. However, this cannot fully explain our results because the mountain time trial, especially the (semi)mountainous sections 1 and 3, did not fluctuate much, that is, it was mainly uphill. Cardiovascular drift might be another explanation.^22^ Heart rate increases up to 15% from 5 to 60 mins of exercise have been reported.^22^ Furthermore, cardiovascular drift is accentuated by factors such as dehydration and heat stress,^22^ which can play a role during a mountain time trial. Nevertheless, HR remains a useful marker to estimate exercise intensity because it displays the body’s response to a given performance.^11^

### Determinants of Finish Time

The PO values measured in the laboratory, especially PO at VT2 and POpeak, were strongly associated with finish time as well as with POmean during the (different sections) of the race. Our results were almost similar to the determinants of a 16-km simulated handcycling time trial,^14^ which took 29 mins on average. The main determinants of the finish time of the simulated race were POpeak and PO at 4 mmol/l, the latter is comparable with our VT2^23^ (both showing an *r* value of −0.77 compared with our *r* value of −0.73 to −0.78, respectively) and aerobic lactate threshold, which is comparable with our VT1^23^ (*r* value of −0.68 compared with our *r* value of −0.64). Other handcycling studies showed even stronger correlations between PO at 4 mmol/l (*r* = 0.927)^13^ and 15-km time trial velocity and between POpeak and 22-km time trial velocity (*r* = 0.85).^24^ These results indicate that both PO at VT2 and POpeak are important indicators of the race time during handcycling races of different durations.

Peak power output showed slightly stronger correlations with POmean during the different race sections than the PO at VT2. The correlation of POpeak with the POmean during the mountainous part was extremely high (*r* = 0.901). Interestingly, the POmean during the flat part of the race showed the strongest correlation with the finish time. Of course, to finish in a good time, it is also important to speed up during the flat parts of a race, which seemed to be the strategy of the WC handcyclist.

### Limitations

Because of the high dropout rate, the results cannot be generalized to the total group of HandbikeBattle participants. The high dropout rate might be due to the low velocities observed during the race in the nonstudy group. A previous cycling study showed that the PowerTap can validly and reliably measure PO even at the lower levels of PO and speed.^25^ However, the lowest cycling speed in that study was 15 km/hr, which is much higher than the average velocity of our nonparticipant group (approximately 5.8 ± 1.7 km/hr). Unfortunately, it was, because of the low sample size, not possible to correct for potential confounders, such as disability or weather conditions.

The WC handcyclist had a different GXT protocol with shorter stage duration above VT2. This could have led to a higher POpeak compared with the trained handcyclists. Last, we unfortunately did not collect information about the weather condition over the race years, neither did we collect information about the handcycles. Both could have impacted the climbing performance compared with the laboratory-based data.

### Practical Implications

The increasing number of people with a disability who are active in recreational and competitive handcycling necessitates scientific knowledge to guide the safe preparation for training and competition. Information about the PO profile during handcycling could be used to develop training programs and to assist in pacing mountainous time trials. Power meters are becoming less expensive and, therefore, will hopefully be more accessible for trained handcyclists in the near future. When power meters become more affordable, future studies might be able to collect PO and HR data during training and competition in a large sample. That would make it feasible to also investigate mechanical and physiological responses in subgroups (e.g., sex or disability) in different conditions (e.g., terrain, weather).

Because the handcyclists cycled most of the race in intensity zones 2 and 3, it is recommended to incorporate these zones also in the training when participating in longer time trials (>20 mins). Especially, training at an intensity around the VT2 might be important because the PO at VT2 is an important determinant of the finish time and is associated strongly with the POmean during the mountain time trial. A previous HandbikeBattle study^26^ described the training intensity distribution in three zones based on the rating of perceived exertion (RPE) during the training period. The intensity of the training was, on average over the group, equally distributed over the three zones, that is, 35% in RPE 1 to 4, 31% in RPE 5 to 6 and 34% in RPE 7 to 10.^26^ This indicates that most of the HandbikeBattle participants also train in the higher intensity zones. Although RPE is, like HR, an internal training load measure, it correlates very well with PO measures during training in most handcyclists.^6^

## CONCLUSIONS

Laboratory outcomes POpeak and PO at VT2 are important performance determinants for longer time trials in handcyclists, and it is, therefore, important to improve these outcomes with training. Because the trained handcyclists cycled most of the race in intensity zones 2 and 3, it is recommended to incorporate these zones also in the training.
